# Associations Between Restrictive Fluid Management and Renal Function and Tissue Perfusion in Adults With Severe Falciparum Malaria: A Prospective Observational Study

**DOI:** 10.1093/infdis/jiz449

**Published:** 2019-08-31

**Authors:** Haruhiko Ishioka, Katherine Plewes, Rajyabardhan Pattnaik, Hugh W F Kingston, Stije J Leopold, M Trent Herdman, Kishore Mahanta, Anita Mohanty, Chandan Dey, Shamsul Alam, Ketsanee Srinamon, Akshaya Mohanty, Richard J Maude, Nicholas J White, Nicholas P J Day, Md Amir Hossain, Md Abul Faiz, Prakaykaew Charunwatthana, Sanjib Mohanty, Aniruddha Ghose, Arjen M Dondorp

**Affiliations:** 1 Mahidol Oxford Tropical Medicine Research Unit, Faculty of Tropical Medicine, Mahidol University, Bangkok, Thailand; 2 Department of Clinical Tropical Medicine, Faculty of Tropical Medicine, Mahidol University, Bangkok, Thailand; 3 Department of Anesthesiology and Critical Care, Jichi Medical University, Saitama Medical Center, Saitama, Japan; 4 Centre for Tropical Medicine and Global Health, Nuffield Department of Clinical Medicine, University of Oxford, Oxford, United Kingdom; 5 Ispat General Hospital, Rourkela, Orissa, India; 6 Chittagong Medical College and Hospital, Chittagong, Bangladesh; 7 Infectious Disease Biology Unit, Research Unit of Institute of Life Sciences, Ispat General Hospital, Rourkela, Orissa, India; 8 Institute of Life Sciences, Department of Biotechnology, Government of India, Bhubaneswar, Orissa, India; 9 Harvard TH Chan School of Public Health, Harvard University, Boston, Massachusetts, USA; 10 Dev Care Foundation, Dhaka, Bangladesh

**Keywords:** acute kidney injury, tissue perfusion, pulmonary edema, restrictive fluid management, severe malaria

## Abstract

**Background:**

Liberal fluid resuscitation has proved harmful in adults with severe malaria, but the level of restriction has not been defined.

**Methods:**

In a prospective observational study in adults with severe falciparum malaria, restrictive fluid management was provided at the discretion of the treating physician. The relationships between the volume of fluid and changes in renal function or tissue perfusion were evaluated.

**Results:**

A total of 154 patients were studied, 41 (26.6%) of whom died. Median total fluid intake during the first 6 and 24 hours from enrollment was 3.3 (interquartile range [IQR], 1.8–5.1) mL/kg per hour and 2.2 (IQR, 1.6–3.2) mL/kg per hour, respectively. Total fluid intake at 6 hours was not correlated with changes in plasma creatinine at 24 hours (n = 116; *r*_s_ = 0.16; *P* = .089) or lactate at 6 hours (n = 94; *r*_s_ = −0.05; *P* = .660). Development of hypotensive shock or pulmonary edema within 24 hours after enrollment were not related to the volume of fluid administration.

**Conclusions:**

Restrictive fluid management did not worsen kidney function and tissue perfusion in adult patients with severe falciparum malaria. We suggest crystalloid administration of 2–3 mL/kg per hour during the first 24 hours without bolus therapy, unless the patient is hypotensive.

## Teaser

The optimum fluid management in severe malaria has not been established. In this prospective observational study, restrictive fluid management did not impair kidney function and tissue perfusion, supporting the safety of this approach in adults with severe falciparum malaria.

Severe falciparum malaria causes multiple organ dysfunction. Metabolic acidosis, coma, anemia, acute kidney injury (AKI), pulmonary edema, and severe jaundice are common manifestations in adult patients [1]. Overall, the case fatality in adults treated with parenteral artesunate is approximately 15%, but this is highly dependent on the number of affected organs and their degree of dysfunction [2]. Adequate supportive treatment is crucial for survival. This includes optimal fluid therapy; however, this has not been well defined. A large, randomized, controlled trial in African children showed that fluid bolus resuscitation in severe malaria with compensated shock markedly increased mortality [3]. A retrospective study in adults with severe malaria showed that fluid loading had no beneficial effect on acid-base status or renal failure [4]. We showed that despite clear indicators of intravascular dehydration, liberal fluid resuscitation guided by transpulmonary thermodilution in adults with severe malaria was not associated with improved renal function or acid-base status, but it did contribute to pulmonary edema, which developed in 36% (10 of 28) of patients [5]. Plasma lactate, as a proxy measure for tissue perfusion, is correlated with microcirculatory obstruction and dysfunction resulting mainly from the sequestration of parasitized red blood cells (RBC) [6, 7]. However, because patients have evidence of intravascular dehydration, failure to administer sufficient fluids could further compromise tissue perfusion and renal function. A recent observational study conducted in Myanmar reported the safety of a conservative fluid regimen in moderately severe falciparum and vivax malaria [8]. Better guidance for fluid therapy in severe malaria is important, in particular in malaria-endemic settings where availability of mechanical ventilation is limited for patients with fluid-induced pulmonary edema or acute respiratory distress syndrome. We hypothesized that restrictive fluid management does not worsen kidney function and tissue perfusion in patients with severe malaria. In this prospective observational study in 3 hospitals in India and Bangladesh, the relationship was assessed between the volume of fluid resuscitation and volume status, progression of AKI, and measures of tissue perfusion. The primary aim was to define better the lower threshold for adequate fluid resuscitation in adult patients with severe falciparum malaria.

## METHODS

### Study Design and Patients

The study was conducted as a part of a prospective observational study on hemodynamics and other pathophysiological aspects of severe falciparum malaria from 2011 to 2016. The study sites were the tertiary referral hospital Chittagong (Chattogram) Medical College Hospital and the subdistrict Ramu Upazila Health Complex in Bangladesh and the Ispat General Hospital (IGH) in Rourkela, India. Eligibility criteria for enrollment were (1) age 16 to 65 years, (2) presence of asexual *Plasmodium falciparum* parasitemia confirmed by peripheral blood microscopy, and (3) severe malaria as defined by modified World Health Organization (WHO) criteria [9], including 1 or more of the following: coma (Glasgow Coma Scale, <11), clinical pulmonary edema (SpO_2_ <90% in ambient air with bilateral crepitation), renal impairment (serum creatinine level ≥2 mg/dL and/or anuria), hypoglycemia (blood glucose level <40 mg/dL), shock (systolic blood pressure [SBP] <80 mmHg with cool extremities), hyperlactatemia (plasma lactate level ≥4 mmol/L), or acidosis (plasma bicarbonate level <15 mmol/L). Patients were excluded if they had documented severe underlying diseases including advanced cardiovascular disease, cirrhosis, or chronic kidney failure. Written informed consent was obtained from patients or attending relatives before enrollment in the study. Ethical approval was obtained from the Ethical Review Committee of Chittagong Medical College, the Institutional Review Board of Ispat General Hospital, and the Oxford Tropical Medicine Research Ethics Committee.

### Patient Management

Patients were treated by the attending physician with intravenous artesunate, and standard supportive care was given according to WHO guidelines [10]. Fluid resuscitation and management were provided according to the clinical judgment of the treating physician based on the local standard of care, which recommends against fluid bolus therapy. Mechanical ventilation and renal replacement therapy (RRT) were started at the discretion of the treating physician.

### Study Procedures

On enrollment, a detailed medical history and physical examination were performed. Peripheral blood thick and thin films for parasite counts were collected at enrollment and then every 6 hours until parasite clearance. Blood gas analysis and plasma lactate measurements were performed every 6 hours until normalization (<2 mmol/L) and then daily, using a handheld automated analyzer (i-STAT; Abbott). Full blood count and routine biochemistry were performed daily. During the first 3 days of admission, fluid intake was recorded every 6 hours, including intravenous fluids, oral fluid consumption, and enteral nasogastric tube feeds; in addition, urine output was recorded every 6 hours. Hemodynamic assessment was by transpulmonary thermodilution (PiCCO-plus; Pulsion Medical Systems) as described previously, and this method was performed by physicians experienced with the technique in patients admitted to the intensive care unit (ICU) [11]. Pregnancy, active bleeding, or thrombocytopenia less than 30 × 10^3^/μL were contraindications for PiCCO-plus assessment. Staging of AKI on enrollment used the Kidney Disease Improving Global Outcome (KDIGO) AKI definitions based on plasma or serum creatinine concentration and urine output [12, 13]. Patients on RRT within 24 hours from enrollment were categorized as AKI stage 3 on admission.

### Statistical Analysis

Differences in baseline demographic characteristics were compared between survivors and fatal cases by Fisher's exact test for categorical variables and Mann-Whitney *U* test or Wilcoxon signed-rank test for continuous variables. Correlations between nonnormally distributed variables were assessed using Spearman's correlation coefficient. The variables associated with plasma lactate level were assessed using multiple linear regression analysis. Where appropriate, data were log transformed. Variables in the final multivariate models were selected on the basis of their significance in the univariate analysis and their biological plausibility on the causal pathway to hyperlactatemia. A 2-sided *P* value of <.05 was considered statistically significant. Analyses were performed using Stata, version 15.0 (StataCorp).

## RESULTS

### Baseline Characteristics

**Figure 1.**
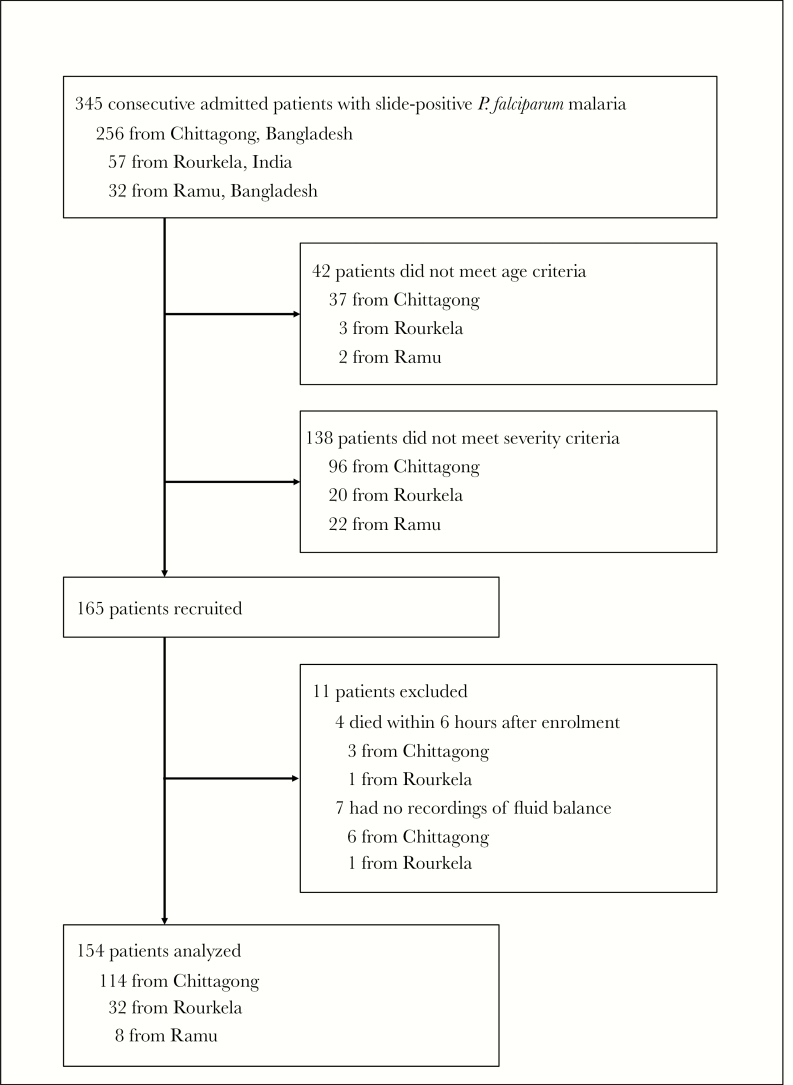
Study profile of patients with falciparum malaria.

Of 345 consecutive patients admitted with slide-positive *P falciparum*, 165 patients with severe falciparum malaria were recruited, 154 of whom were analyzed (Figure 1). Case fatality overall was 41 of 154 (26.6%); 19 patients died within 24 hours of enrollment (Table 1).

**Table 1.** Baseline Characteristics of Patients With Severe Falciparum Malaria Stratified by Outcome^a^

Variables		Survivors		Fatal Cases		Total	*P* Value^b^
	n	113	n	41	n	154	
Age, year		30 [24–40]		32 [24–40]		30 [24–40]	.648
Male gender, n (%)		79 (69.9)		20 (48.8)		99 (64.3)	.022
Clinical Measures							
Duration of fever before admission	112	7 [5–9]		7 [6–9]	153	7 [5–9]	.686
Temperature, °C		37.9 [37.1–38.9]	40	38.4 [37.3–39.3]	153	38.0 [37.1–38.9]	.187
Respiratory rate/minute		28 [24–36]		38 [30–44]		30 [26–40]	<.001
Pulse rate/minute		104 [91–122]		121 [106–134]		109 [94–124]	<.001
Systolic blood pressure, mmHg	112	109 [99–119]		110 [102–124]	153	110 [100–120]	.349
Diastolic blood pressure, mmHg	112	69 [60–75]	40	67 [55–78]	152	68 [58–76]	.927
Glasgow coma scale		10 [8–15]		8 [5–10]		9 [8–14]	<.001
Laboratory Measures							
White cell count, ×10^3^/μL	109	8.9 [6.4–10.9]	40	12.0 [9.3–20.2]	149	9.2 [7.0–12.7]	<.001
Hemoglobin, g/dL		9.0 [7.0–11.2]		8.6 [6.2–11.4]		8.9 [6.9–11.2]	.424
Platelets, ×10^3^/μL	102	36 [22–53]	40	30 [18–44]	142	33 [22–53]	.100
Sodium, mmol/L	112	137 [132–142]		137 [131–145]	153	137 [131–142]	.798
Potassium, mmol/L	112	4.0 [3.5–4.6]		4.2 [3.8–5.3]	153	4.0 [3.6–4.8]	.050
Chloride, mmol/L	112	106 [103–112]		109 [103–112]	153	107 [103–112]	.419
Glucose, mg/dL	109	121 [98–155]		103 [89–148]	150	118 [95–155]	.239
Blood urea nitrogen, mg/dL	110	37 [22–72]		50 [35–98]	151	42 [25–77]	.029
Creatinine, mg/dL		1.5 [1.0–3.2]		2.0 [1.4–3.5]		1.7 [1.1–3.4]	.039
Total bilirubin, mg/dL	112	2.2 [1.1–5.0]		6.6 [1.5–17.1]	153	2.6 [1.1–7.0]	.044
pH	112	7.405 [7.357–7.449]	40	7.375 [7.289–7.421]	152	7.390 [7.347–7.440]	.018
Base deficit, mEq/L	112	6 [4–10]	40	12 [7–16]	152	7 [4–11]	<.001
HCO_3_, mmol/L	112	19.2 [16.2–20.8]	40	14.1 [10.7–18.0]	152	17.9 [14.7–20.4]	<.001
Lactate, mmol/L	111	2.6 [1.9–4.7]	40	5.3 [3.5–9.0]	151	3.0 [2.1–5.4]	<.001
Parasitological Measures							
Parasitemia, /μL		40 694 [3480–203 472]		75 109 [5024–228 466]		44 896 [4000–217 790]	.439
PfHRP2, ng/mL	90	2727 [1513–5874]	37	3427 [1280–12 440]	127	2918 [1421–7201]	.504
Severity of Diseases							
No. of severity criteria on enrollment, n		1 [1–2]		3 [2–3]		2 [1–2]	<.001
Cerebral malaria, GCS <11, n (%)		69 (61.1)		34 (82.9)		103 (66.9)	.012
Renal impairment, creatinine ≥2.0 mg/dL, n (%)		50 (44.3)		22 (53.7)		72 (46.8)	.362
Hypoglycemia, glucose <40 mg/dL, n (%)		2 (1.8)		1 (2.4)		3 (2.0)	1.000
Hypotension, SBP <80 mmHg, n (%)		1 (0.9)		2 (4.9)		3 (1.9)	.173
Pulmonary edema, n (%)		2 (1.8)		4 (9.8)		6 (3.9)	.043
Metabolic acidosis, HCO_3_ <15 mmol/L, n (%)		19 (16.8)		22 (53.7)		41 (26.6)	<.001
Hyperlactatemia, lactate ≥4 mmol/L, n (%)		37 (32.7)		27 (65.9)		64 (41.6)	<.001
Blood transfusion, n (%)		55 (48.7)		12 (29.3)		67 (43.5)	.043
Mechanical ventilation, n (%)		4 (3.5)		15 (36.6)		19 (12.3)	<.001
Renal replacement therapy, n (%)		32 (28.3)		13 (31.7)		45 (29.2)	.692

Abbreviations: GCS, Glasgow Coma Scale; HCO_3_, bicarbonate; IQR, interquartile range; PfHRP2; *Plasmodium falciparum* histidine-rich protein 2; SBP, systolic blood pressure.

^a^All data as median [IQR], except where otherwise indicated. The number of cases (n) in each group are 113, 41, and 154, respectively, except where indicated. Fisher's exact test for categorical variables, Mann-Whitney *U* test for continuous variables.

^b^Comparison between fatal cases and survivors.

### Fluid Balance

The median intravenous fluid volume administered was 3.0 (IQR, 1.7–4.6) mL/kg per hour during the first 6 hours after enrollment (n = 151) (Table 2). Including oral or enteral intake, median total fluid intake was as follows: 3.3 (IQR, 1.8–5.1) mL/kg per hour, range 0.3–16.7 mL/kg per hour (n = 151) in the first 6 hours; and 2.2 (IQR, 1.6–3.2) mL/kg per hour, range 0.5–6.8 mL/kg per hour (n = 127) in the first 24 hours. Approximately 90% of fluids administered were crystalloids, including isotonic fluids (0.9% NaCl, 0.9% NaCl/5% dextrose) and hypotonic fluids (Ringer's lactate, 0.45% NaCl/5% dextrose). The remainder included 5% or 10% dextrose solutions and whole blood. Thirty-six patients received blood transfusion during first 24 hours with a median volume of 0.36 (range 0.04–1.47) mL/kg per hour. No colloid intravenous solutions were used. The median cumulative fluid balance was +730 (range −1350 to +3750) mL in the first 6 hours (n = 143) and +1595 (range −2500 to +5745) mL in the first 24 hours (n = 110). To explore potential drivers of differences in the administered fluid volumes by the treating physicians, we constructed a multivariate model with the total volume administered at 6 hours as dependent variable and enrollment SBP, heart rate, body temperature, delayed capillary refill time, plasma creatinine, blood urea nitrogen level, and duration of fever before admission as independent variables (n = 145). Of these, only SBP on enrollment was independently associated with the total fluid intake in the first 6 hours (β, −0.351; *P* < .001; *R*^2^ = 0.14).

**Table 2.** Intravenous Fluid Administration, Total Fluid Intake, Urine Output, and Fluid Balance Over the First 24 Hours of the Study^a^

Time	Intravenous			Total			Urine Output			In/Out Balance	
	n	mL	mL/kg per hour	n	mL	mL/kg per hour	n	mL	mL/kg per hour	n	mL
First 6 hour	151	1000 [510–1600]	3.0 [1.7–4.6]	151	1000 [600–1700]	3.3 [1.8–5.1]	144	300 [73–500]	0.9 [0.2–1.6]	143	730 [300–1473]
First 12 hour	150	1530 [1000–2500]	2.5 [1.5–3.8]	150	1700 [1100–2500]	2.7 [1.7–3.9]	125	550 [190–910]	0.9 [0.3–1.4]	124	1065 [550–1905]
First 24 hour	130	2652 [1750–3400]	2.1 [1.2–2.9]	127	3000 [2060–3900]	2.2 [1.6–3.2]	111	1190 [470–1910]	1.0 [0.4–1.5]	110	1595 [725–2425]

^a^All data as median [interquartile range]. Total included intravenous fluid and oral intake or enteral feeding. The amount of the fluid is cumulative volume.

### Global End-Diastolic Volume Index

Transpulmonary thermodilution data were available from 9 patients admitted to ICU, 4 (44%) of whom had a fatal outcome. Median global end-diastolic volume index (GEDVI) at first measurement was 560 (IQR, 505–629) mL/m^2^ (Table 3), well below the reference range for normovolemia (680–800 mL/m^2^). There was no difference in initial GEDVI between fatal cases and survivors (median 692 vs 524 mL/m^2^; *P* = .086). Initial GEDVI had no correlation with plasma creatinine or lactate concentration on enrollment (*r*_s_ = 0.40, *P* = .284 and *r*_s_ = 0.67, *P* = .071, respectively). The median change in GEDVI was −7 (IQR, −54 to 26) mL/m^2^ after 6 hours and −15 (IQR, −48 to 2) mL/m^2^ after 12 hours, whereas the median total fluid intake over the same period was 2.0 (IQR, 1.7–2.9) mL/kg per hour and 2.4 (IQR, 1.9–4.0) mL/kg per hour, respectively.

**Table 3.** Hemodynamic and Pulmonary Measures Until 24 Hours After Initial Measurement^a^

Variables	Initial Measurement	6 Hours	12 Hours	Reference Ranges
	n = 9	n = 9	n = 9	
Hemodynamic Measures				
CI, dyne-sec/cm^5^/m^2^	3.69 [2.99–4.75]	3.78 [3.57–4.85]	3.75 [3.38–4.47]	3.5–5.0
GEDVI, mL/m^2^	560 [505–629]	543 [533–555]	537 [455–581]	680–800
SVRI, dyne-sec/cm^5^/m^2^	1514 [1311–2171]	1367 [1324–1767]	1766 [1187–1826]	1700–2400
Pulmonary Measures				
EVLWI, mL/kg	6.62 [6.35–6.94]	6.23 [6.25–7.69]	6.46 [6.12–8.29]	3.0–7.0
PVPI	1.67 [1.53–2.03]	1.50 [1.43–1.90]	1.80 [1.47–2.20]	1.0–3.0

Abbreviations: CI, cardiac index; EVLWI, extravascular lung water index; GEDVI, global end-diastolic volume index; PVPI, pulmonary vascular permeability index; SVRI, systemic vascular resistance index.

^a^All data as median [interquartile range].

### Fluid Administration and Progression of Acute Kidney Injury

On enrollment, 118 patients (76.6%) had AKI, 56 (47.5%) of whom had KDIGO stage 3; 45 (29.2%) patients received RRT, 26 of whom received RRT within 24 hours. Among 118 patients with complete follow-up, 53 (44.9%) had an increase in plasma creatinine at 24 hours (median 0.72 mg/dL; IQR, 0.30–1.80 mg/dL) (Supplement 1). The median total fluid intake in these patients was 2.2 mL/kg per hour (IQR, 1.6–2.9 mL/kg per hour) during the first 24 hours (n = 50) compared to 2.1 mL/kg per hour (IQR, 1.5–3.3 mL/kg per hour) in patients without an increase in creatinine (n = 61) (*P* = .843). Volumes of fluids at 6, 12, or 24 hours were not correlated with a change in plasma creatinine at 24 hours. After excluding 21 of 118 patients who received RRT within 24 hours, there was no correlation between total fluid intake during the first 6 hours and a change in creatinine at 24 hours (n = 95; *r*_s_ = 0.02; *P* = .884). A similar result was observed with fluid intake during the first 12 or 24 hours. Urine output during the first 6, 12, or 24 hours was significantly correlated with a change in creatinine at 24 hours (n = 88, *r*_s_ = −0.24, *P* = .025; n = 82, *r*_s_ = −0.37, *P* < .001; and n = 81, *r*_s_ = −0.35, *P* = .001, respectively), whereas the cumulative fluid balance during the first 6, 12, or 24 hours was not (n = 88, *r*_s_ = 0.17, *P* = .108; n = 82, *r*_s_ = 0.12, *P* = .275; and n = 81, *r*_s_ = 0.22, *P* = .052, respectively).

### Fluid Administration and Plasma Lactate

Among 96 patients with follow-up lactate data, 32 of 96 (33.3%) had an increase in plasma lactate at 6 hours (median 0.79 mg/dL; IQR, 0.29–1.04 mg/dL). The median total fluid intake in these patients was 3.3 (IQR, 2.0–4.4) mL/kg per hour during the first 6 hours (n = 31) compared to 2.6 (IQR, 1.4–4.3) mL/kg per hour in patients without an increase in lactate (n = 63) (*P* = .155). There was no correlation between the total fluid intake during the first 6 hours and change in plasma lactate at 6 hours (n = 94, *r*_s_ = −0.05, *P* = .660). A multiple linear regression model on 71 patients with complete data on potential contributors to hyperlactatemia (Table 4) showed that a shock episode within 6 hours from enrollment, enrollment hemoglobin, and enrollment *P falciparum* histidine-rich protein 2 (PfHRP2) were independently associated with the proportional change in plasma lactate at 6 hours. Total fluid intake in the first 6 hours and study site did not contribute to the model.

**Table 4.** Multiple Linear Regression Analysis of Variables With Proportional Change in Plasma Lactate Concentrations^a^

Variable	β Value	*P* Value
PfHRP2^b^	0.194	.037
Parasitemia^b^	−0.101	.369
Hemoglobin	−0.302	.006
Mean blood pressure	0.122	.236
Shock within 6 hours	0.530	<.001
Total fluid intake during 6 hours	−0.134	.550
R square	0.446	
Adjusted R square	0.395	

Abbreviations: PfHRP2, *Plasmodium falciparum* histidine-rich protein 2.

^a^The regression model was based on 71 patients.

^b^Variable was analyzed on a log scale.

### Incidence of Shock

Three of 154 (1.9%) patients had hypotensive shock on enrollment. Another 19 of 151 patients (12.6%) developed clinically significant shock defined by a SBP <80 mmHg with cool extremities or vasopressor use after enrollment: 12 within 24 hours and another 3 during the following 24 hours. Microbiological culture facilities were not available, but concomitant bacterial infections were probable contributing factors: 1 patient had a septic abortion and 4 patients were diagnosed with hospital-acquired infections, 2 of which had aspiration pneumonia. In the 12 patients with an onset of the shock within 24 hours, median time to the onset was 12.3 (IQR, 7.3–15) hours and 11 of 12 (91.7%) died after a median 8.0 (IQR, 2.5–20.0) hours from the onset of shock. Median total fluid intake until the onset of shock was 5.0 (IQR, 2.4–6.3) mL/kg per hour, ranging from 1.7 to 14.0 mL/kg per hour (n = 12). Among them, 1 patient had pulmonary edema on enrollment and developed shock after 6.5 hours and a total fluid intake of 5.1 mL/kg per hour. Another patient developed shock and pulmonary edema at 12 hours after receiving a total fluid volume of 4.8 mL/kg per hour. The median total fluid intake during the first 6 hours in 12 patients developing shock was 4.4 (IQR, 2.6–6.2) mL/kg per hour compared to 3.3 (IQR, 1.8–5.0) mL/kg per hour in 129 patients without shock throughout admission (*P* = .167).

### Incidence of Pulmonary Edema

Clinical pulmonary edema was defined as bilateral crepitation on auscultation combined with an oxygen saturation less than 90% on ambient air or equivalent saturations corrected for supplemented oxygen. A total of 6 of 154 (3.9%) had clinical pulmonary edema at the time of enrollment, and 21 of 148 (14.2%) developed pulmonary edema after enrollment. Chest x-ray confirmed the diagnosis in 6 cases, but this was not available for the remainder. Mechanical ventilation was available in 11 of 27 (40.7%) patients with pulmonary edema. The mortality rate in this group was 17 of 27 (63.0%). The median time from enrollment to newly developed pulmonary edema was 24 (IQR, 9–46) hours, and 11 of 21 (52.4%) developed pulmonary edema within 24 hours. The median total fluid intake until the onset of pulmonary edema was 3.7 (IQR, 1.3–7.3) mL/kg per hour, ranging from 0.9 to 8.9 mL/kg per hour (n = 11). Three patients suffered an episode of shock preceding pulmonary edema, receiving a total fluid intake of 8.3 mL/kg per hour over 5 hours, 7.3 mL/kg per hour over 6 hours, and 3.3 mL/kg per hour over 24 hours, respectively, before developing pulmonary edema.

## DISCUSSION

This observational study from resource-limited settings evaluated the current local practice of conservative fluid management in patients with strictly defined severe falciparum malaria. Restrictive intravenous fluid administration (median 3.0 mL/kg per hour; IQR, 1.7–4.6 mL/kg per hour) during the first 6 hours did not result in worsening of kidney function or hyperlactatemia. The volume of administered fluids did not predict the development of circulatory shock within 24 hours after enrollment. Fluid volumes also did not predict development of pulmonary edema in this setting. The incidence of pulmonary edema (14%) was much lower than observed in our earlier study (31%) in the same setting evaluating liberal fluid resuscitation, with a median fluid administration of 227 mL/hour during 24 hours (range 30–572 mL/hour) [5]. The recommended fluid management in severe malaria has varied with changing concepts of pathophysiology. In the 1980s a cautious approach summarized as “keep the patient on the dry side” was advocated [14–16]. Previous researchers argued that the 2 infectious processes of severe malaria and bacterial sepsis had similar cytokine-mediated pathologies [17, 18], and recommendations for more aggressive fluid replacement regained traction [19]. These recommendations were reversed in children, when the results of the definitive FEAST study indicated that this approach increased mortality [3]. The Surviving Sepsis Campaign guidelines in patients with sepsis-induced tissue hypoperfusion and suspicion of hypovolemia recommend an initial fluid challenge of minimal 30 mL/kg crystalloids [20]. The observations from our study in combination with earlier fluid studies in severe malaria confirm the suggestion that this recommendation is not applicable to patients with severe malaria and hyperlactatemia in the absence of circulatory shock. Restricted fluid therapy is applicable despite intravascular hypovolemia, as also observed in a subset of patients in this study with transpulmonary thermodilution measurements and in other studies describing hypovolemia in both adults and children with severe malaria using various techniques [5, 21]. In our study, admission or change in GEDVI, as a measure of hypovolemia, did not correlate with admission or change in plasma lactate; the volume of fluid resuscitation also did not correlate with the change of plasma lactate in the acute phase of the disease. It should be noted that early resolution of hyperlactatemia is a strong surrogate for patient survival in severe malaria [22]. These results suggest that in the absence of severe hypotension, hypovolemia is not an important factor limiting tissue perfusion, and that liberal fluid resuscitation does not improve tissue perfusion in severe malaria. More likely, hyperlactatemia results from a compromised microcirculation caused by sequestration of parasitized RBC and reduced RBC deformability, erythrocyte agglutination, and endothelial dysfunction [23–27]. In our study, hemoglobin, a determinant of oxygen delivery, was independently associated with the proportional change in plasma lactate at 6 hours, suggesting a potential benefit of blood transfusion.

Despite intravascular dehydration and a proinflammatory response in severe malaria, hypotension is not frequently observed [2, 28]. Bacterial coinfection is one of the causes of hypotension in severe malaria, Bloodstream bacterial coinfection is common in pediatric severe malaria [1]. A recent study from Myanmar reported a high incidence of concomitant bacteremia in adults with falciparum malaria [29]. The low incidence of hypotensive shock in severe malaria relates to preserved systemic vascular resistance and cardiac function possibly related to high concentrations of plasma cell-free hemoglobin [5], and it is compatible with our transpulmonary thermodilution findings in the current study. This contrasts with bacterial sepsis, where hypotensive shock is more common, despite the much lower pathogen density in the blood [30]. Consequently, hypotensive shock is an infrequent indication for fluid bolus therapy in severe malaria, whereas the presence of hypotension should raise the suspicion of concomitant bacterial sepsis.

Acute kidney injury is a common complication in adult severe malaria with an incidence between 20% and 40% [1, 2]. The pathogenesis of malaria-associated AKI is incompletely understood, but it includes reduced microcirculatory flow resulting from sequestration of parasitized RBC in glomerular and tubulointerstitial vessels, tubular oxidative damage through free hemoglobin, and inflammatory changes [31]. Despite intravascular hypovolemia, our earlier study showed that aggressive fluid resuscitation in hypovolemic patients with severe malaria did not affect progression of AKI [5]. The current study shows that restrictive fluid administration does not worsen kidney function, suggesting a limited contribution of intravascular hypovolemia to malaria-associated AKI.

World Health Organization guidelines on the management of adult severe malaria recommend restrictive fluid management of 3–5 mL/kg per hour with 0.9% normal saline during the first 6 hours, followed by 2–3 mL/kg per hour of 0.5% dextrose/0.9% normal saline [32]. Of note is that restrictive fluid management was the usual practice in the participating centers. This is partly driven by the limited access to mechanical ventilation, making fluid overload induced pulmonary edema a feared complication. Our results support this approach but suggest that an even more restricted regimen of 2–3 mL/kg per hour of crystalloids during the first 6 hours without bolus therapy may be preferable. A limitation of the current study is its observational design. However, testing different fluid regimens in a randomized trial is difficult: using large fluid volumes have proven harmful, and with less contrasting interventions between study arms, only a very large trial will be sufficiently powered. This is challenging because of the decreasing burden of adult severe malaria in low transmission settings [33]. Our relatively small sample size, another limitation of the current study, is also a reflection of the decrease in malaria transmission in the region.

## CONCLUSIONS

In conclusion, our study suggests that a restrictive fluid management does not worsen kidney function, tissue perfusion, or blood pressure and might reduce the incidence of pulmonary edema in adult patients with severe falciparum malaria. Further larger studies are warranted to confirm the safety and efficacy of the restrictive fluid approach. From the current data, we suggest administration of 2–3 mL/kg per hour crystalloids fluids during the first 24 hours without bolus therapy, unless the patient is hypotensive.

## Supplementary Data

Supplementary materials are available at *The Journal of Infectious Diseases* online. Consisting of data provided by the authors to benefit the reader, the posted materials are not copyedited and are the sole responsibility of the authors, so questions or comments should be addressed to the corresponding author.

jiz449_suppl_Supplementary_Material

## Notes

***Acknowledgments.*** We thank the patients and their relatives for participating in these studies. We also thank research assistants, attending physicians, and the staff at Chittagong (Chattogram) Medical College Hospital, Ispat General Hospital, and Ramu Upazilla Health Complex for their support.

***Financial support.*** This work was funded by the Wellcome Trust as part of the Wellcome Trust Major Overseas Programme and the Faculty of Tropical Medicine, Mahidol University.

***Potential conflicts of interest.*** All authors: No reported conflicts of interest. All authors have submitted the ICMJE Form for Disclosure of Potential Conflicts of Interest.

Presented in part: 1st Malaria World Congress, July 4, 2018, Melbourne, Australia.
