# Supplementary material for: Associations Between Restrictive Fluid Management and Renal Function and Tissue Perfusion in Adults With Severe Falciparum Malaria: A Prospective Observational Study
Source: J Infect Dis. 2019 Aug 31;221(2):285–92. doi: 10.1093/infdis/jiz449 (PMC6935998; doi:10.1093/infdis/jiz449)
Supplement: jiz449_suppl_Supplementary_Material [file jiz449_suppl_supplementary_material.docx]

Supplement 1. Severity of acute kidney injury and fluid intake stratified by creatinine increase

| Variables |  | Patients with creatinine increase at 24 hours |  | Patients without creatinine increase at 24 hours | P-value |
| --- | --- | --- | --- | --- | --- |
|  | n | 53 | n | 65 |  |
| Creatinine on enrollment, mg/dL |  | 2.0 [1.2-3.3] |  | 1.4 [1.0-3.2] | 0.415 |
| AKI stage on enrollment |  |  |  |  |  |
| no AKI, n (%) |  | 13 (24.5) |  | 13 (20.0) |  |
| Stage 1, n (%) |  | 8 (15.1) |  | 16 (24.6) |  |
| Stage 2, n (%) |  | 7 (13.2) |  | 17 (26.2) |  |
| Stage 3, n (%) |  | 25 (47.2) |  | 19 (29.2) |  |
| Renal replacement therapy |  |  |  |  |  |
| Total, n (%) |  | 24 (45.3) |  | 14 (21.5) | 0.010 |
| within 24 hours, n (%) |  | 7 (29.2)^a^ |  | 14 (100.0)^a^ |  |
| after 24 hours, n (%) |  | 17 (70.8)^a^ |  | 0 (0)^a^ |  |
| Fluid intake |  |  |  |  |  |
| during 6 hours, mL/kg/hour | 52 | 3.4 [1.9-4.4] | 64 | 2.5 [1.6-5.4] | 0.280 |
| during 12 hours, mL/kg/hour |  | 2.7 [1.7-3.6] | 64 | 2.6 [1.6-3.7] | 0.906 |
| during 24 hours, mL/kg/hour | 50 | 2.2 [1.6-2.9] | 61 | 2.1 [1.5-3.3] | 0.843 |

All data as median [IQR], except where otherwise indicated.

^a^ proportion to the patients who underwent renal replacement therapy.
